# Brain-acting hepatokines: its impact on energy balance and metabolism

**DOI:** 10.3389/fnins.2025.1589110

**Published:** 2025-05-15

**Authors:** Lucía Giovanini, Nahuel Wanionok, Mario Perello, Maria Paula Cornejo

**Affiliations:** ^1^Laboratory of Neurophysiology of the Multidisciplinary Institute of Cell Biology [IMBICE, Argentine Research Council (CONICET) and Scientific Research Commission, Province of Buenos Aires (CIC-PBA), National University of La Plata], La Plata, Buenos Aires, Argentina; ^2^Department of Surgical Sciences, Functional Pharmacology and Neuroscience, University of Uppsala, Uppsala, Sweden

**Keywords:** hepatokines, brain, metabolism, energy balance, liver

## Abstract

The liver is recognized for its central role in energy metabolism, yet emerging evidence highlights its function as an endocrine organ, secreting a variety of proteins—hepatokines—that influence distant tissues. Hepatokines not only regulate metabolic processes by acting on peripheral tissues but also exert direct effects on brain function. In this mini-review, we discuss the existing literature on the role of “brain-acting” hepatokines including IGF-1, FGF21, LEAP2, GDF15, and ANGPTLs, and their impact on energy balance and metabolism. We review the existing evidence regarding their roles in metabolism through their action in the brain, and their potential implications in metabolic disturbances. By integrating insights from recent studies, we aim to provide a comprehensive understanding of how liver-derived signals can modulate energy balance and metabolism.

## Introduction

**Figure 1:**
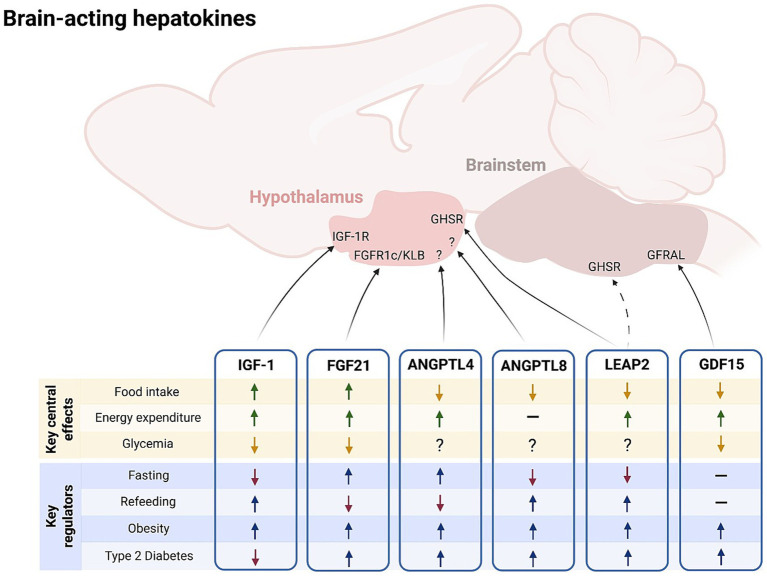
Brain-acting hepatokines: key central effects and key regulators. Liver-derived hepatokines FGF21, IGF-1, LEAP2, GFD15, ANGPTL4 and ANGPTL8 act on the central nervous system to modulate energy balance and metabolism (key central effects). Circulating levels of these brain-acting hepatokines vary according to the energetic status and in certain metabolic disorders (key regulators). ANGPTL, angiopoietin-like protein; FGF21, fibroblast growth factor 21; GDF15, growth differentiation factor 15; IGF-1, insulin-like growth factor 1; LEAP2, liver-expressed antimicrobial peptide 2. Figure created under the license of BioRENDER (https://BioRender.com/zelorai, license number: SH285C03YT).

Energy metabolism encompasses the complex biochemical pathways by which organisms extract, convert, and store energy from nutrients such as lipids, carbohydrates, and proteins. The energy obtained from nutrients is expended in numerous physiological processes (i.e., resting, activity-induced and diet-induced energy expenditure), and the difference between the energy obtained and the energy expended makes up energy balance. When the amount of energy obtained continuously surpasses the amount expended, there is an excessive storage of energy that ends with an increase in body weight, leading to overweight and obesity. In recent decades, the world has witnessed an alarming rise in the prevalence of overweight and obesity. These conditions are hallmarked by persistent energy imbalance and profound metabolic dysregulation, and they now represent major public health challenges. Obesity, in particular, is a well-established risk factor for a spectrum of metabolic diseases, most notably type 2 diabetes mellitus (T2DM; Schnurr et al., 2020) and metabolic-associated steatotic liver disease (MASLD; Younossi et al., 2016). While lifestyle and environmental factors play critical roles, growing evidence highlights the contribution of endogenous signals in the development and maintenance of these metabolic disorders. Among these, liver-derived peptides—collectively known as hepatokines—have recently emerged as key regulators of systemic metabolism (Zhang et al., 2023). Not only do hepatokines influence peripheral tissues, but a subset also communicates directly with the brain to modulate metabolism, appetite, and energy expenditure. Here, we aim to synthesize current knowledge on brain-acting hepatokines (Figure 1), emphasizing their emerging roles as metabolic integrators and potential therapeutic targets in the context of obesity and related disorders.

## Liver-to-brain communication

The liver plays a central role in regulating multiple aspects of energy metabolism, acting as a metabolic hub that coordinates nutrient processing, glycogen storage, lipid synthesis, glucose and lipid homeostasis, and hormonal signaling in response to feeding and fasting. The liver also has a predominant endocrine function since up to 40% of liver transcripts encode secreted peptides with multiple regulatory functions (Uhlén et al., 2015). Hepatocytes, the main cellular type of the liver, produce and secrete a variety of peptides called hepatokines, which serve as key mediators through which the liver communicates metabolic information to distant tissues, including the brain. In Table 1, we listed the main recognized hepatokines, indicated their receptors or target systems and organs, and also cited evidence implicating each hepatokine in the modulation of metabolism and energy balance.

Hepatokines are one of the ways by which the liver and the brain communicate. The liver also receives sympathetic and parasympathetic innervations that allow the brain to regulate liver function, and also the liver to convey metabolic information to the brain. Of note, multisynaptic brain projections to the liver comprise hypothalamic and medullary brain regions (Stanley et al., 2010), and sensory information from the hepatic portal vein reaches medullary brain centers through vagal afferents (Zsombok et al., 2024; Garcia-Luna et al., 2021). Interestingly, pharmacogenetic and optogenetic manipulation of specific hypothalamic neuronal populations modulates the expression of hepatic enzymes involved in glucose homeostasis (Kwon et al., 2020; Coutinho et al., 2017).

**Table 1:** Metabolic implications of hepatokines.

Hepatokine	Receptor/Target system	Target organs	Metabolic implications	
			In rodent models	In humans
Activin E (inhibin subunit beta E)	Heterotetrameric complex of activin receptor IIA and receptor-like kinase 7 (ALK7)	Liver and adipose tissue	Suppresses lipolysis (Griffin et al., 2023) Stimulates energy expenditure and increases insulin sensitivity (Hashimoto et al., 2018) Positive correlation between expression of activin E gene and insulin resistance (Sekiyama et al., 2019)	Loss-of-function mutations in the activin E gene contribute to healthier fat distribution (Deaton et al., 2022) Correlation between increased expression of activin E gene expression and insulin resistance (Sugiyama et al., 2018)
Adropin	G protein-coupled receptor 19 (GPR19) Notch1 ligand NB3/Contactin6 (CNTN6)	Brain, liver, adipose tissue, muscle, cardiovascular system	Adropin-overexpressing mice or systemic adropin treatment attenuates hepatic steatosis and insulin resistance in DIO (Kumar et al., 2008) Adropin-deficient mice exhibited dyslipidemia and insulin resistance (Gao et al., 2014) Adropin administrations improve glucose homeostasis and protect against DIO (Gao et al., 2019)	Lower adropin levels in T2DM patients (Zang et al., 2018) and in patients with MASLD (Kutlu et al., 2019)
ANGPTL3	Lipoprotein lipase (LPL) activity modulation through ANGPTL3-4-8 complex Different receptors according to cell type	Liver, adipose tissue, skeletal muscle, and cardiac tissue	ANGPTL3-overexpressing mice show increased plasma triglycerides, cholesterol and non-esterified fatty acids (Koishi et al., 2002)	Positive correlation between circulating ANGPTL3 and plasma glucose, insulin, and HOMA-IR levels in patients with insulin resistance (Jensen-Cody and Potthoff, 2020) Loss-of-function mutations in the ANGPTL3 gene produce lower plasma LDL cholesterol and triglycerides (Kersten, 2021)
ANGPTL4 (fasting-induced adipose factor (FIAF))	Lipoprotein lipase (LPL) activity modulation through ANGPTL3-4-8 complex Integrin α5/β1 and α5β3 (keratinocytes)	Brain, liver, adipose tissue, skeletal muscle	ANGPTL4-deficient mice have lower plasma triglycerides, whereas ANGPTL4-overexpressing mice show increased plasma triglycerides (Sylvers-Davie and Davies, 2021)	ANGPTL4 deficiency is associated with higher circulating HDL cholesterol levels (Sylvers-Davie and Davies, 2021) Loss-of-function mutation in ANGPTL4 gene results in lower plasma triglycerides and higher HDL cholesterol (Olshan and Rader, 2018)
ANGPTL6 (angiopoietin-related growth factor (AGF))	Not described	Liver, adipose tissue, skeletal muscle	ANGPTL6-overexpressing mice show enhanced insulin sensitivity (Oike et al., 2005) ANGPTL6-deficient mice show impaired insulin sensitivity and decreased energy expenditure (Ebert et al., 2014)	Higher ANGPTL6 plasma levels in T2DM patients (Ebert et al., 2014) and in obese individuals (Qaddoumi et al., 2020) Increased serum ANGPTL6 levels precedes the development of metabolic syndrome (Namkung et al., 2011)
ANGPTL8 (lipasin or betatrophin)	Lipoprotein lipase (LPL) activity modulation through ANGPTL3-4-8 complex Leukocyte immunoglobulin-like receptor B (LILRB3, cardiomyocytes)	Brain, liver, adipose tissue, skeletal muscle, cardiac tissue	ANGPTL8-deficient mice show disrupted triglycerides metabolism (Wang et al., 2018) ANGPTL8-overexpressing mice show increased plasma triglycerides (Zhang, 2012)	Positive correlation between serum ANGPTL8 levels and the severity of MASLD (Lee et al., 2016) Controversial results on plasma ANGPTL8 levels and obesity (Luo and Peng, 2018)
Fetuin A (Alpha-2-HS-glycoprotein)	Insulin receptor	Liver, adipose tissue, skeletal muscle	Fetuin A-deficient mice show improved insulin sensitivity and resistance to DIO (Mathews et al., 2002)	SNPs in fetuin A gene are associated with T2DM (Mathews et al., 2002; Andersen et al., 2008) Increased circulating fetuin A levels in humans with a high liver fat content
FGF21	Heterocomplex of FGF receptor 1c (FGFR1c) and the co-receptor Klotho-β (KLB)	Brain and adipose tissue	Decreases blood glucose levels and improves insulin sensitivity in diabetic rodents (Kharitonenkov et al., 2005) FGF21-deficient mice show lower blood glucose levels (Liang et al., 2014) FGF21-overexpressing mice show reduced body weight and plasma insulin levels (Owen et al., 2014)	Increased FGF21 plasma levels in individuals with obesity, insulin resistance, MASLD and T2DM (Zhang et al., 2008; Ďurovcová et al., 2010; Dushay et al., 2010; Cheng et al., 2011) Treatment with FGF21 analog improves dyslipidemia, decreases body weight and improves fasting insulin levels in patients with T2DM and obesity (Gaich et al., 2013)
Follistatin	Binds and neutralizes activins	Liver, adipose tissue, pancreas, skeletal muscle, brain, placenta	Follistatin-overexpressing mice show a decrease in abdominal fat content, increased glucose clearance, and improved plasma lipid profiles (Singh et al., 2017) Follistatin treatment promotes white adipose tissue browning (Li et al., 2019)	Increased plasma follistatin levels in T2DM patients (Hansen et al., 2013; Sylow et al., 2020) Higher hepatic follistatin expression in MASLD patients (Tong et al., 2022) Moderate increase in plasma follistatin levels in obese patients (Maïmoun et al., 2020)
GDF15 (macrophage inhibitory cytokine-1)	Glial-derived neurotrophic factor receptor alpha-like (GFRAL)	Brain, adipose tissue, skeletal muscle	GDF15 treatment decreases food intake and body weight (Hale and Véniant, 2021) GDF15-deficient mice show increased food intake and body weight, and impaired glucose tolerance (Wang et al., 2021) GDF15-overexpressing mice have diminished food intake and body weight, and also improved insulin sensitivity (Wang et al., 2021)	Higher plasma GDF15 in obese (Hale and Véniant, 2021) and in MASLD patients (Koo et al., 2018)
Hepassocin (fibrinogen-like protein 1 or hepatocyte-derived fibrinogen-related protein 1)	Epidermal growth factor receptor (EGFR)	Liver, adipose tissue, skeletal muscle	Hepatic overexpression of hepassocin induces hepatic steatosis, whereas hepassocin deletion improves high fat diet-induced hepatic steatosis (Wu et al., 2013) Hepassocin treatment improves liver functions in diabetic mice (Ou et al., 2017)	Increased hepassocin plasma levels in MASLD subjects (Wu et al., 2013) Higher plasma hepassocin levels in obese individuals (Huang et al., 2020) Increased plasma hepassocin in patients with T2DM (Abdelmoemen et al., 2019)
IGF-1	IGF1 receptor (IGF1R)	Skeletal muscle, brain	Animal models with reduced plasma IGF-1 show increased circulating GH, leading to hyperinsulinemia, lower insulin sensitivity, and impaired carbohydrate metabolism (Yakar et al., 2001; Yakar et al., 2004)	Higher circulating free IGF-1 in obese patients (Yakar et al., 2001; Frystyk et al., 1995) Altered plasma levels of IGF-BPs and free IGF-1 in T2DM patients (Clemmons, 2018)
LEAP2	Growth hormone secretagogue receptor (GHSR)	Brain, pituitary, pancreas	LEAP2 plasma levels are increased in DIO and in diabetic mice (Mani et al., 2019; Lugilde et al., 2022) LEAP2 treatment reduces food intake and feeding-induced increase of blood glucose (Ge et al., 2018; Hagemann et al., 2022)	LEAP2 treatment reduces food intake in an *ad libitum* meal and lowers postprandial plasma glucose and growth hormone concentrations (Hagemann et al., 2022) Increased plasma LEAP2 levels in obese subjects (Holm et al., 2022; Mani et al., 2019; Andreoli et al., 2024) and in T2DM patients (Li et al., 2022)
LECT2	Endothelial cell-specific orphan receptor (Tie1) CD209 antigen-like protein A (CD209a) Tyrosine kinase with immunoglobulin-like and EGF-like domains 1 (Tie1) Tyrosine protein kinase Met (MET) L1 cell adhesion molecule (L1CAM) Transferrin (Trf)	Skeletal muscle, liver, adipose tissue, endothelium	LECT2-deficient mice show increased insulin sensitivity (Lan et al., 2014) Circulating LECT2 levels correlate with hepatic triglycerides and responds to dietary changes preceding body weight changes (Chikamoto et al., 2016)	Circulating LECT2 correlates with the severity of obesity and insulin resistance (Lan et al., 2014) Increased plasma LECT2 levels in patients with MASLD and metabolic syndrome (Yoo et al., 2017) Increased circulating LECT2 in T2DM patients and negative correlation with HDL cholesterol in diabetic and obese subjects (Zhang et al., 2018)
Lipocalin 13	Binds to small hydrophobic molecules (fatty acids, phospholipids, steroids, among others)	Adipose tissue and liver	Contradictory results on the involvement of lipocalin 13 in glucose and lipid homeostasis (Cho et al., 2011; Sheng et al., 2011; Bühler et al., 2021)	Human LCN13 has not been identified yet
MANF	Neuroplastin receptor	Brain, liver, pancreas, adipose tissue, heart, kidney	MANF-deficient mice show increased blood glucose levels, decreased plasma insulin levels and lower body weight (Lindahl et al., 2014; Pakarinen et al., 2020; Danilova et al., 2019) MANF-overexpressing mice show higher energy expenditure and enhanced browning of inguinal adipose tissue (Wu et al., 2021) MANF administration retards body weight gain and improves glucose homeostasis in obese mice (Tang et al., 2022)	Elevated circulating MANF levels in prediabetic and T2DM patients (Wu et al., 2021; Fu et al., 2021) Plasma MANF levels reduced in MASLD patients (Sousa-Victor et al., 2019) Inconsistent results on circulating MANF levels in obesity (Tang et al., 2022)
Selenoprotein P	Apolipoprotein E receptor-2 (ApoER2) Megalin (lipoprotein receptor-related protein 2, LRP2) Lipoprotein receptor-related protein 1 (LRP1)	Liver, skeletal muscle, small intestine, colon, spleen, testis, kidney	Selenoprotein P treatment induces glucose intolerance and insulin resistance, whereas hepatic knockdown of selenoprotein P improved glucose intolerance and insulin resistance (Misu et al., 2010)	Increased plasma selenoprotein P levels in T2DM patients (Misu et al., 2010; Yang et al., 2011) Increased concentrations of selenoprotein P decrease the risk of metabolic syndrome (Gharipour et al., 2017)
SHBG	SHBG receptor	Liver, testis	SHBG-overexpressing mice gain less body weight under a high fat diet (Saez-Lopez et al., 2020)	SNPs in SHBG gene associated with increased risk of T2DM (Ding et al., 2009) Lower plasma SHBG levels in MASLD patients and associated with high-grade MASLD in patients with T2DM (Shin et al., 2011) Lower plasma SHBG levels in obese individuals (Cooper et al., 2015)
SMOC 1	Regulates cell-matrix interactions by binding to laminins, C-reactive protein, and tenascin-C	Liver, skeletal muscle	SMOC1 treatment improved glycemic control in lean mice without changes in insulin secretion (Montgomery et al., 2020)	Lower plasma SMOC1 levels in insulin-resistant individuals (Montgomery et al., 2020) No evidence that plasma SMOC1 levels are causally associated with T2DM, MASLD, and glycemic traits (Ghodsian et al., 2021)
Tsukushi	Modulates Wnt, transcription growth factor beta (TGF-β), cell communication network factor (CCN2/CTGF), and netrin signaling pathways	Liver, adipose tissue	Controversial results regarding tsukushi effects on energy balance (Mouchiroud et al., 2019a) and its involvement in MASLD rodent models (Xiong et al., 2019; Mouchiroud et al., 2019b)	Lower tsukushi plasma levels in obese individuals (Li et al., 2021) Higher circulating levels of tsukushi in subjects with metabolic syndrome (Li et al., 2023) Higher plasma Tsukushi levels in individuals with MASLD and positive correlation with the degree of liver steatosis and fibrosis (Lam et al., 2024)

ANGPTL, angiopoietin-like protein; DIO, diet-induced obesity; FGF21, fibroblast growth factor 21; GDF15, growth differentiation factor 15; HDL, high density lipoprotein; HOMA-IR, homeostatic model assessment for insulin resistance; GH, growth hormone; IGF1, insulin-like growth factor 1; LDL, low density lipoprotein; LEAP2, liver-expressed antimicrobial peptide 2; LECT2, Leukocyte cell-derived chemotaxin 2; MANF, mesencephalic astrocyte-derived neurotrophic factor; MASLD, metabolic-associated steatotic liver disease; SHBG, sex hormone-binding globulin; SMOC 1, SPARC related modular calcium binding 1; SNPs, single-nucleotide polymorphisms; T2DM, type 2 diabetes mellitus.

Hepatokines are constitutively secreted into circulation and reach distant tissues, including the brain, to modulate multiple aspects of metabolism (Schulze et al., 2019). Like other circulating factors, hepatokines can access the brain through distinct mechanisms: by crossing the blood–brain barrier, by diffusing through fenestrated capillaries in specialized brain regions, or by crossing the blood-cerebrospinal fluid (CSF) barrier constituted by hypothalamic tanycytes and choroid plexus cells (Rawal et al., 2022). In the brain, two essential hubs in the control of metabolism are the hypothalamus and the medulla. In the hypothalamus, the arcuate nucleus (ARH) plays a pivotal role in sensing circulating factors and transmitting peripheral information to other hypothalamic and extra-hypothalamic nuclei. The ARH comprises different neuronal subtypes (Campbell et al., 2017), including those co-expressing neuropeptide Y (NPY) and agouti-related peptide (AgRP), and neurons co-expressing pro-opiomelanocortin (POMC) and cocaine-and amphetamine-regulated transcript. Together with the ARH, the hypothalamic paraventricular (PVH), dorsomedial and ventromedial nuclei, and also the lateral hypothalamic area are all interconnected and form a neural network with key regulatory roles in metabolism (Jais and Brüning, 2022). In the medulla, the dorsal vagal complex (DVC)–comprising the dorsal motor nucleus of the vagus, the area postrema (AP), and the nucleus of the solitary tract (NTS)–contains multiple neuronal populations that are crucial for interpreting and relaying peripheral signals to the hypothalamus, and also to elicit autonomic responses via the vagus nerve (Abdalla, 2017). Emerging evidence suggests that hepatokines can act on these brain regions directly or indirectly, influencing central pathways that control metabolism, food intake, and energy expenditure.

## Brain-acting hepatokines and metabolism

### Insulin-like growth factor 1 (IGF-1)

IGF-1 is a peptide with structural homology to insulin produced in the liver, where its production is stimulated by growth hormone (GH), and is strongly implicated in the modulation of cell growth and differentiation. Plasma IGF-1 is bound to one of the six IGF-1 binding proteins (IGF-1BP), which modulate its effects. IGF-1 production is regulated by nutrient availability, with hepatic IGF-1 mRNA and plasma levels declining with fasting and increasing with refeeding (Straus and Takemoto, 1990; Clemmons, 2012). IGF-1 mainly signals through IGF-1 receptor (IGF-1R), which is ubiquitously expressed in peripheral organs and the brain. The net effect of IGF-1 is determined by the modulation of IGF-1 production, IGF-1BPs levels, and IGF-1R expression. We recommend consulting in-depth reviews for a comprehensive understanding of these regulatory mechanisms (Yuen et al., 2024; Clemmons, 2018). For instance, fasting decreases plasma IGF-1 levels in healthy subjects (Rahmani et al., 2019), whereas altered plasma IGF-1BPs levels are observed in obese patients, producing a subtle increase in free IGF-1 (Clemmons, 2012). Also, disturbed levels of circulating IGF-BPs and free IGF-1 are detected in T2DM patients, with changes depending on the progression of the pathology (Clemmons, 2018).

IGF-1 is produced in the brain during development, and some studies have shown that central IGF-1 modulates energy metabolism and energy balance in adulthood. Intra-cerebro-ventricular (ICV) IGF-1 injection lowers hepatic glucose production in hyperinsulinemic-clamped mice (Muzumdar et al., 2006) and improves insulin sensitivity of aged rats (Huffman et al., 2016). ICV IGF-1 treatment in mice increases food intake and enhances insulin sensitivity (Hong et al., 2017). Moreover, deletion of IGF-1R from kisspeptin-expressing neurons produces a decrease in food intake and an increase in energy expenditure exclusively in female mice (Wang et al., 2024). However, partial deletion of neuronal IGF-1R causes subtle metabolic phenotypes, including higher circulating triglycerides and free fatty acids and a moderate hyperglycemia (Kappeler et al., 2008). Thus, the complexity of the IGF-1 system makes it difficult to dissect a specific modulatory role for central IGF-1 on energy balance and metabolism.

### Fibroblast growth factor 21 (FGF21)

FGF21 belongs to the fibroblast growth factor family, a group of proteins involved in regulating multiple processes such as angiogenesis and embryonic development. FGF21 decreases blood glucose and improves insulin sensitivity in diabetic rodents (Kharitonenkov et al., 2005). FGF21 mRNA levels increase in the liver of high-sucrose diet consuming and diet-induced obese (DIO) mice (Fisher et al., 2010; Maekawa et al., 2017). Circulating FGF21 levels increase in fasted mice (Markan et al., 2014), in mice chronically fed high-sucrose diets (Maekawa et al., 2017), in DIO mice (Fisher et al., 2010), and in a mouse model of T2DM (Spolcová et al., 2014). In humans, circulating FGF21 increases during fasting (Fazeli et al., 2015). Also, circulating FGF21 is increased in overweight/obese individuals (Zhang et al., 2008; Ďurovcová et al., 2010), and in patients with MASLD (Dushay et al., 2010) and T2DM (Cheng et al., 2011), and in women with gestational diabetes (Tan et al., 2013). Moreover, plasma levels of FGF21 increase after gastric sleeve surgery (Al-Regaiey et al., 2024), whereas a decrease is detected in individuals with obesity/overweight and MASLD after weight loss (Erdem et al., 2024).

FGF21 is primarily produced in the liver and regulates energy balance and metabolism by acting on the brain, particularly in the hypothalamus (Hsuchou et al., 2007; Liang et al., 2014). In humans, FGF21 was detected in the CSF (Tan et al., 2013; Tan et al., 2011; Li et al., 2016). FGF21 acts via the FGF receptor 1c (FGFR1c), which is widely distributed in the mouse brain, whereas its co-receptor Klotho-*β* (KLB, (Ogawa et al., 2007)) is specifically present in the hypothalamic suprachiasmatic nucleus, the DVC, and also in the amygdala (Bookout et al., 2013; Bono et al., 2022; Claflin et al., 2022). KLB genetic deletion in hypothalamic neurons abolishes the reductions in body weight and plasma insulin observed in FGF21-overexpressing mice (Bookout et al., 2013). Moreover, hypothalamic KLB deletion blunts the increase in food consumption and energy expenditure and the decrease in plasma glucose and cholesterol observed in DIO FGF21-overexpressing mice (Owen et al., 2014). Interestingly, pharmacogenetic activation of KLB-expressing neurons increases energy expenditure and decreases body weight of DIO mice, and genetic deletion of KLB from glutamatergic neurons prevents FGF21 effects on energy expenditure and body weight (Claflin et al., 2022).

Studies performing central infusions of FGF21 also demonstrate its central effects. Chronic ICV injection of FGF21 improves insulin sensitivity of lean and DIO rats (Sarruf et al., 2010), whereas FGF21 ICV administrations lower body weight, percent body fat, and plasma glucose and cholesterol concentrations of DIO mice, effects dependent on hypothalamic KLB expression (Owen et al., 2014). Interestingly, ICV FGF21 treatment induces the expression of thermogenic genes and also increases sympathetic nerve activity in brown adipose tissue of DIO mice, both effects dependent on hypothalamic KLB expression (Owen et al., 2014). Moreover, ICV FGF21 administration to hypoglycemic FGF21-deficient mice normalizes their glycemia, which depends on the presence of FGFR1c in the PVH (Liang et al., 2014). Furthermore, ICV FGF21 administrations increase ERK1/2 expression in the mouse hypothalamus (Yang et al., 2012), whereas intra-PVH FGF21 injection increases ERK1/2 and CREB phosphorylation (Liang et al., 2014). Thus, the literature indicates that FGF21 impacts and depends on the activity of hypothalamic brain nuclei to modulate energy balance and metabolism, which supports the notion of FGF21 as a brain-acting hepatokine.

### Liver-expressed antimicrobial peptide 2 (LEAP2)

LEAP2 was recently described as a ligand of the GH secretagogue receptor (GHSR) (Ge et al., 2018; M’Kadmi et al., 2019), which triggered a great interest on this hepatokine (Perelló, 2025). LEAP2 mRNA is predominantly found in liver hepatocytes and jejunal enterocytes of both mice and humans (Ge et al., 2018; Krause et al., 2003; Englund et al., 2024). LEAP2 is secreted via the constitutive secretory pathway, thereby its production is controlled at the gene expression level, with metabolic status as a key regulatory factor (Perelló, 2025). Liver LEAP2 mRNA levels decrease with fasting (Holm et al., 2022; Islam et al., 2020), and increase in DIO mice (Holm et al., 2022). Interestingly, liver LEAP2 mRNA decreases in mice fed with a ketogenic diet (Holm et al., 2022), whereas it increases in mice orally administered with glucose and corn oil (Islam et al., 2024). Circulating LEAP2 levels in mice decrease with fasting (Ge et al., 2018; Islam et al., 2020; Mani et al., 2019; Fernandez et al., 2022), whereas plasma LEAP2 is increased in DIO (Mani et al., 2019; Holá et al., 2023; Casado et al., 2024) and *ob/ob* mice (Lugilde et al., 2022), and in a mouse model of type 1 diabetes mellitus (T1DM) (Mani et al., 2019). Interestingly, LEAP2-overexpressing mice show enhanced body weight loss and impaired maintenance of glycemia under caloric restriction (Ge et al., 2018). Conversely, female LEAP2-deficient mice display increased food intake and enhanced body weight gain when fed a high fat diet (HFD) (Shankar et al., 2021). In humans, obese adults and children show increased plasma LEAP2 levels (Holm et al., 2022; Mani et al., 2019; Andreoli et al., 2024; Fittipaldi et al., 2020), which positively correlate with body mass index, percentage of body fat, and homeostatic model assessment of insulin resistance, among other parameters (Mani et al., 2019; Stark et al., 2023). Circulating LEAP2 levels are also increased and positively correlate with glycosylated hemoglobin in T2DM patients (Li et al., 2022). Interestingly, decreased plasma LEAP2 levels are detected in healthy men after exercise (Holm et al., 2022) and also in obese subjects after a short calorie restriction (Ragland and Malin, 2023).

Studies performing LEAP2 administrations demonstrate its ability to modulate energy balance. LEAP2 blunts fasting-induced food intake (Holá et al., 2022), and also diminishes food intake-induced increase in blood glucose in mice and humans (Hagemann et al., 2022). Interestingly, lipidized analogs of LEAP2 also display anorexigenic effects (Holá et al., 2022). The central effect of LEAP2 has also been addressed. ICV LEAP2 administration decreases HFD consumption of mice in a binge-like eating protocol (Cornejo et al., 2019), and also diminishes fasting-induced and spontaneous intake in rats and mice (Lugilde et al., 2022; Tufvesson-Alm et al., 2023). Chronic central administration of LEAP2 diminishes food intake and weight gain (Chu et al., 2022) and also reduces circulating triglycerides, while increasing energy expenditure and thermogenic biomarkers in brown adipose tissue of mice (Casado et al., 2024). To the best of our knowledge, only few studies have addressed the putative neuronal population mediating LEAP2 effects on food intake. LEAP2 was shown to hyperpolarize ARH NPY-expressing neurons, preventing their activation by ghrelin (Mani et al., 2019). Accordingly, LEAP2-deficient mice show enhanced ghrelin-induced ARH activation, measured as c-Fos immunoreactivity (Shankar et al., 2021), whereas food-deprived mice ICV administered with LEAP2 show decreased fasting-induced c-Fos in the ARH (Fernandez et al., 2022). Moreover, chronic central LEAP2 administration increases c-Fos immunoreactivity in ARH POMC neurons and chemogenetic inhibition of POMC neurons blunts LEAP2’s anorexigenic effect (Chu et al., 2022). Thus, experimental evidence demonstrates the ability of LEAP2 to modulate the activity of different hypothalamic neuronal populations, including ARH NPY and POMC neurons, which may mediate its effect on energy balance and metabolism.

### Growth differentiation factor 15 (GDF15)

GDF15 is a divergent member of the transforming growth factor *β* superfamily. GDF15 mRNA is detected in multiple human and mouse tissues (Lockhart et al., 2020). Liver GDF15 mRNA levels are increased in *ob/ob* mice and Zucker diabetic fatty rats (Xiong et al., 2017), and also in DIO mice (Patel et al., 2019). Genetically modified rodent models have helped to understand the role of GDF15 on the regulation of energy balance. GDF15-deficient mice show increased food intake and body weight, and impaired glucose tolerance (Wang et al., 2021). Moreover, mice with genetic deletion of glial-derived neurotrophic factor receptor alpha-like (GFRAL), GDF15’s receptor, show attenuated DIO and insulin resistance (Emmerson et al., 2017; Mullican et al., 2017; Hsu et al., 2017; Yang et al., 2017). Conversely, GDF15-overexpressing mice have diminished food intake and body weight, and improved insulin sensitivity (Wang et al., 2021). In humans, increased liver mRNA and plasma levels of GDF15 are found in individuals with MASLD (Koo et al., 2018), whereas circulating GDF15 is also increased in obese and T2DM patients (Wang et al., 2021).

GFRAL mRNA is selectively detected in the mouse and human hindbrain (Mullican et al., 2017; Hes et al., 2025). Central GDF15 administration diminishes food intake and induces c-Fos expression in the AP and NTS (Tsai et al., 2014; Worth et al., 2020), and, although the identity of GDF15-responding neurons remains elusive, GFRAL expression co-localizes with cholecystokinin-expressing neurons (Worth et al., 2020). Interestingly, systemic GDF15 treatment diminishes gastric emptying in rodents, an effect dependent on the vagal efferent pathway (Xiong et al., 2017), and also causes emesis in musk shrews (Borner et al., 2020). Furthermore, GDF15 induces taste aversion (Patel et al., 2019; Worth et al., 2020), an effect consistent with c-Fos induction in the amygdala of GDF15-administered mice (Hsu et al., 2017). Thus, the effects of GDF15 as a brain-acting hepatokine that regulates energy balance seem to be secondary to its effect on other processes such as gastric emptying or taste aversion, processes that rely on different central circuits.

### Angiopoietin-like protein 4 (ANGPTL4)

ANGPTL4 belongs to the ANGPTL protein family, which are involved in angiogenesis. ANGPTL4 is produced in the liver and adipose tissue, and, to a lesser extent, in the pituitary and hypothalamus (Wiesner et al., 2004; Kim et al., 2010; Vienberg et al., 2015). In mice, liver and plasma ANGPTL4 peptide levels increase with fasting and decrease after refeeding (Kim et al., 2010). Interestingly, ANGPTL4 peptide levels—but not mRNA levels—increase in the hypothalamus of fasted mice (Wiesner et al., 2004; Kim et al., 2010), likely due to elevated circulating peptide. Hypothalamic ANGPTL4 mRNA is increased in mouse models of T1DM and T2DM (Vienberg et al., 2015). ANGPTL4-deficient mice show reduced plasma triglycerides when fasted (Köster et al., 2005), whereas they show enhanced body weight gain and visceral adipose tissue mass deposit, but improved glucose tolerance when fed a HFD (Janssen et al., 2018). Strikingly, deletion of ANGPTL4 from hepatocytes enhances plasma triacylglycerol clearance and insulin sensitivity, and also diminishes weight gain of DIO mice (Singh et al., 2021). Conversely, ANGPTL4-overexpressing mice show increased serum cholesterol and triglycerides (Köster et al., 2005). In humans, circulating ANGPTL4 is increased in T2DM patients (Babapoor-Farrokhran et al., 2015; McCulloch et al., 2020) and in obese individuals (Schinzari et al., 2021) and to decrease, together with body weight and fat mass, after bariatric surgery (Bini et al., 2022).

Only one study links the central effect of ANGPTL4 to energy balance (Kim et al., 2010). ICV ANGPTL4 suppresses fasting-induced hyperphagia and increases energy expenditure, effects absent with peripheral administration (Kim et al., 2010). Moreover, ICV ANGPTL4 decreases hypothalamic AMPK phosphorylation, and the pharmacologic inhibition of AMPK signaling blunts the effect of ANGPTL4 on food intake (Kim et al., 2010). Then, ANGPTL4’s role as a brain-acting hepatokine needs further work to confirm its central effect on energy balance, although it seems evident that ANGPTL4’s effect relies on hypothalamic circuits.

### Angiopoietin-like protein 8 (ANGPTL8)

ANGPTL8 is another member of the ANGPTL protein family and is exclusively produced in the human liver, whereas its mRNA is detected in mouse liver and adipose tissue (Zhang, 2012). In mice, ANGPTL8 liver mRNA levels decrease with fasting and increase with refeeding (Quagliarini et al., 2012), HFD feeding (Zhang, 2012), and also in hyperinsulinemic (Zhang et al., 2020) and fatty liver mice (Lee et al., 2016). Circulating and CSF levels of ANGPTL8 are also higher in diabetic murine models (Meng et al., 2024). Liver ANGPTL8 overexpression increases serum triacylglycerol and very-low density lipoprotein concentrations (Cox et al., 2015), whereas ANGPTL8 deficiency produces a decrease in plasma triglycerides and improves glucose tolerance of DIO mice (Zhang et al., 2020). In humans, circulating ANGPTL8 levels decrease with fasting and increase with refeeding (Quagliarini et al., 2012). Plasma ANGPTL8 levels are also increased in individuals with T1DM (Espes et al., 2014), T2DM (Espes et al., 2014; Fu et al., 2014; Hu et al., 2014) and obesity (Fu et al., 2014), and positively correlate with hepatic steatosis in MASLD patients (von Loeffelholz et al., 2017). Interestingly, a recent study showed that ANGPTL8 CSF levels are increased in T2DM patients with cognitive dysfunction (Meng et al., 2024).

Only one study addressed ANGPTL8’s central modulation of energy balance in mice. ICV ANGPTL8 administration decreases rebound feeding and body weight regain after fasting, whereas it affects fasting-induced c-Fos in hypothalamic nuclei (Wang et al., 2018). Also, chronic ICV ANGPTL8 administration decreases body weight and circulating free fatty acids (Wang et al., 2018). Thus, further studies supporting the modulatory effect of ANGPTL8 on hypothalamic brain nuclei are needed to unequivocally establish its role as a brain-acting hepatokine.

## Concluding remarks

Hepatokines have emerged as key players not only in peripheral metabolism but also in the complex regulation of brain function and whole-body energy balance. In this mini-review, we introduced the concept of “brain-acting hepatokines”, those that impact on the activity of brain centers that control energy balance and metabolism. Although the current understanding of the specific roles of brain-acting hepatokines in the central nervous system remains uneven, accumulating evidence underscores their relevance in both health and disease. Their dual function—as metabolic messengers and potential therapeutic targets—highlights the urgency and promise of advancing research in this field. Furthermore, since plasma levels of brain-acting hepatokines are increased in pathological conditions implicating metabolic disturbances such as T2DM, MASLD, and obesity, their potential as biomarkers poses them as putative targets for early diagnosis, prevention, and monitoring of disease progression. Moreover, the possibility of a synergistic central effect of brain-acting hepatokines has been only incipiently studied. Continued exploration of hepatokine signaling pathways may unlock novel strategies to address metabolic disorders and neuroendocrine dysregulation.

## Glossary

AgRP: agouti-related peptide
AMPK: AMP-activated protein kinase
ANGPTL4: angiopoietin-like protein 4
ANGPTL8: angiopoietin-like protein 8
AP: area postrema
ARH: hypothalamic arcuate nucleus
CSF: cerebrospinal fluid
DIO: diet-induced obese
DVC: dorsal vagal complex
FGF-21: fibroblast growth factor 21
FGFR1c: FGF receptor 1c
GDF15: growth differentiation factor 15
GFRAL: glial-derived neurotrophic factor receptor alpha-like
GH: growth hormone
GHSR: GH secretagogue receptor
HFD: high fat diet
ICV: intra-cerebro-ventricular
IGF-1: insulin-like growth factor 1
IGF-1BP: IGF-1 binding proteins
IGF-1R: IGF-1 receptor
KLB: Klotho-*β*
LEAP2: liver-expressed antimicrobial peptide 2
MASLD: metabolic-associated steatotic liver disease
NPY: neuropeptide Y
NTS: nucleus of the solitary tract
POMC: pro-opiomelanocortin
PVH: paraventricular hypothalamic nucleus
T1DM: type 1 diabetes mellitus
T2DM: type 2 diabetes mellitus

## References

[ref1] Abdalla M. M. I. (2017). Central and peripheral control of food intake. Endocr. Regul. 51, 52–70. doi: 10.1515/enr-2017-0006, PMID: 28222022

[ref2] Abdelmoemen G. Khodeir S. A. Zaki A. N. Kassab M. Abou-Saif S. Abd-Elsalam S. (2019). Overexpression of Hepassocin in diabetic patients with nonalcoholic fatty liver disease may facilitate increased hepatic lipid accumulation. Endocr. Metab. Immune Disord. Drug Targets 19, 185–188. doi: 10.2174/1871530318666180716100543, PMID: 30009716

[ref3] Al-Regaiey K. A. Iqbal M. Alzaid M. A. Alkaoud O. A. Alhadyani M. A. Alagel O. A.. (2024). Evaluating fibroblast growth factor 21 (FGF21) levels post-gastric sleeve surgery in obese patients. Cureus. 16:e66122. doi: 10.7759/cureus.66122, PMID: 39100807 PMC11298160

[ref4] Andersen G. Burgdorf K. S. Sparsø T. Borch-Johnsen K. Jørgensen T. Hansen T.. (2008). AHSG tag single nucleotide polymorphisms associate with type 2 diabetes and dyslipidemia: studies of metabolic traits in 7,683 white Danish subjects. Diabetes 57, 1427–1432. doi: 10.2337/db07-0558, PMID: 18316360

[ref5] Andreoli M. F. Fittipaldi A. S. Castrogiovanni D. De Francesco P. N. Valdivia S. Heredia F.. (2024). Pre-prandial plasma liver-expressed antimicrobial peptide 2 (LEAP2) concentration in humans is inversely associated with hunger sensation in a ghrelin independent manner. Eur. J. Nutr. 63, 751–762. doi: 10.1007/s00394-023-03304-8, PMID: 38157050

[ref6] Babapoor-Farrokhran S. Jee K. Puchner B. Hassan S. J. Xin X. Rodrigues M.. (2015). Angiopoietin-like 4 is a potent angiogenic factor and a novel therapeutic target for patients with proliferative diabetic retinopathy. Proc. Natl. Acad. Sci. USA 112, E3030–E3039. doi: 10.1073/pnas.1423765112, PMID: 26039997 PMC4466723

[ref7] Bini S. D’Erasmo L. Astiarraga B. Minicocci I. Palumbo M. Pecce V.. (2022). Differential effects of bariatric surgery on plasma levels of ANGPTL3 and ANGPTL4. Nutr. Metab. Cardiovasc. Dis. 32, 2647–2654. doi: 10.1016/j.numecd.2022.08.019, PMID: 36163215 PMC10018753

[ref8] Bono B. S. Koziel Ly N. K. Miller P. A. Williams-Ikhenoba J. Dumiaty Y. Chee M. J. (2022). Spatial distribution of beta-klotho mRNA in the mouse hypothalamus, hippocampal region, subiculum, and amygdala. J. Comp. Neurol. 530, 1634–1657. doi: 10.1002/cne.25306, PMID: 35143049

[ref9] Bookout A. L. de Groot M. H. M. Owen B. M. Lee S. Gautron L. Lawrence H. L.. (2013). FGF21 regulates metabolism and circadian behavior by acting on the nervous system. Nat. Med. 19, 1147–1152. doi: 10.1038/nm.3249, PMID: 23933984 PMC3769420

[ref10] Borner T. Shaulson E. D. Ghidewon M. Y. Barnett A. B. Horn C. C. Doyle R. P.. (2020). GDF15 induces anorexia through nausea and Emesis. Cell Metab. 31, 351–362.e5. doi: 10.1016/j.cmet.2019.12.004, PMID: 31928886 PMC7161938

[ref11] Bühler L. Maida A. Vogl E. S. Georgiadi A. Takacs A. Kluth O.. (2021). Lipocalin 13 enhances insulin secretion but is dispensable for systemic metabolic control. Life Sci. Alliance 4. doi: 10.26508/lsa.202000898, PMID: 33536239 PMC7898469

[ref12] Campbell J. N. Macosko E. Z. Fenselau H. Pers T. H. Lyubetskaya A. Tenen D.. (2017). A molecular census of arcuate hypothalamus and median eminence cell types. Nat. Neurosci. 20, 484–496. doi: 10.1038/nn.4495, PMID: 28166221 PMC5323293

[ref13] Casado S. Varela-Miguéns M. de Oliveira D. T. Quintela-Vilariño C. Nogueiras R. Diéguez C.. (2024). The effects of ghrelin and LEAP-2 in energy homeostasis are modulated by thermoneutrality, high-fat diet and aging. J. Endocrinol. Investig. 47, 2061–2074. doi: 10.1007/s40618-024-02307-4, PMID: 38337094 PMC11266414

[ref14] Cheng X. Zhu B. Jiang F. Fan H. (2011). Serum FGF-21 levels in type 2 diabetic patients. Endocr. Res. 36, 142–148. doi: 10.3109/07435800.2011.558550, PMID: 21973233

[ref15] Chikamoto K. Misu H. Takayama H. Kikuchi A. Ishii K. A. Lan F.. (2016). Rapid response of the steatosis-sensing hepatokine LECT2 during diet-induced weight cycling in mice. Biochem. Biophys. Res. Commun. 478, 1310–1316. doi: 10.1016/j.bbrc.2016.08.117, PMID: 27562717

[ref16] Cho K. W. Zhou Y. Sheng L. Rui L. (2011). Lipocalin-13 regulates glucose metabolism by both insulin-dependent and insulin-independent mechanisms. Mol. Cell. Biol. 31, 450–457. doi: 10.1128/MCB.00459-10, PMID: 21135134 PMC3028623

[ref17] Chu G. Peng H. Yu N. Zhang Y. Lin X. Lu Y. (2022). Involvement of POMC neurons in LEAP2 regulation of food intake and body weight. Front. Endocrinol. 13:932761. doi: 10.3389/fendo.2022.932761, PMID: 36387867 PMC9650057

[ref18] Claflin K. E. Sullivan A. I. Naber M. C. Flippo K. H. Morgan D. A. Neff T. J.. (2022). Pharmacological FGF21 signals to glutamatergic neurons to enhance leptin action and lower body weight during obesity. Mol. Metab. 64:101564. doi: 10.1016/j.molmet.2022.101564, PMID: 35944896 PMC9403559

[ref19] Clemmons D. R. (2012). Metabolic actions of insulin-like growth factor-I in normal physiology and diabetes. Endocrinol. Metab. Clin. N. Am. 41, 425–443. doi: 10.1016/j.ecl.2012.04.017, PMID: 22682639 PMC3374394

[ref20] Clemmons DR. (2018). 40 YEARS OF IGF1: Role of IGF-binding proteins in regulating IGF responses to changes in metabolism. Available online at: https://jme.bioscientifica.com/view/journals/jme/61/1/JME-18-0016.xml (Accessed March 3, 2025). 10.1530/JME-18-0016 29563157

[ref21] Cooper L. A. Page S. T. Amory J. K. Anawalt B. D. Matsumoto A. M. (2015). The association of obesity with sex hormone-binding globulin is stronger than the association with ageing – implications for the interpretation of total testosterone measurements. Clin. Endocrinol. 83, 828–833. doi: 10.1111/cen.12768, PMID: 25777143 PMC4782930

[ref22] Cornejo M. P. Castrogiovanni D. Schiöth H. B. Reynaldo M. Marie J. Fehrentz J.. (2019). Growth hormone secretagogue receptor signalling affects high-fat intake independently of plasma levels of ghrelin and LEAP 2, in a 4-day binge eating model. J. Neuroendocrinol. 31:e12785. doi: 10.1111/jne.12785, PMID: 31469195

[ref23] Coutinho E. A. Okamoto S. Ishikawa A. W. Yokota S. Wada N. Hirabayashi T.. (2017). Activation of SF1 neurons in the ventromedial hypothalamus by DREADD technology increases insulin sensitivity in peripheral tissues. Diabetes 66, 2372–2386. doi: 10.2337/db16-1344, PMID: 28673934

[ref24] Cox A. R. Lam C. J. Bonnyman C. W. Chavez J. Rios J. S. Kushner J. A. (2015). Angiopoietin-like protein 8 (ANGPTL8)/betatrophin overexpression does not increase beta cell proliferation in mice. Diabetologia 58, 1523–1531. doi: 10.1007/s00125-015-3590-z, PMID: 25917759 PMC4473078

[ref25] Danilova T. Galli E. Pakarinen E. Palm E. Lindholm P. Saarma M.. (2019). Mesencephalic astrocyte-derived neurotrophic factor (MANF) is highly expressed in mouse tissues with metabolic function. Front Endocrinol 10:765. doi: 10.3389/fendo.2019.00765/full PMC6851024 31781038

[ref26] Deaton A. M. Dubey A. Ward L. D. Dornbos P. Flannick J. Yee E.. (2022). Rare loss of function variants in the hepatokine gene INHBE protect from abdominal obesity. Nat. Commun. 13:4319. doi: 10.1038/s41467-022-31757-8, PMID: 35896531 PMC9329324

[ref27] Ding E. L. Song Y. Manson J. E. Hunter D. J. Lee C. C. Rifai N.. (2009). Sex hormone-binding globulin and risk of type 2 diabetes in women and men. N. Engl. J. Med. 361, 1152–1163. doi: 10.1056/NEJMoa0804381, PMID: 19657112 PMC2774225

[ref28] Ďurovcová V. Marek J. Hána V. Matoulek M. Zikán V. Haluzíková D.. (2010). Plasma concentrations of fibroblast growth factors 21 and 19 in patients with Cushing’s syndrome. Physiol. Res. 59, 415–422. doi: 10.33549/physiolres.931801, PMID: 19681655

[ref29] Dushay J. Chui P. C. Gopalakrishnan G. S. Varela-Rey M. Crawley M. Fisher Ffolliott M.. (2010). Increased fibroblast growth factor 21 in obesity and nonalcoholic fatty liver disease. Gastroenterology 139, 456–463. doi: 10.1053/j.gastro.2010.04.054, PMID: 20451522 PMC4862867

[ref30] Ebert T. Kralisch S. Loessner U. Jessnitzer B. Stumvoll M. Fasshauer M.. (2014). Relationship between serum levels of angiopoietin-related growth factor and metabolic risk factors. Horm. Metab. Res. 46, 685–690. doi: 10.1055/s-0034-1382078, PMID: 25011017

[ref31] Emmerson P. J. Wang F. Du Y. Liu Q. Pickard R. T. Gonciarz M. D.. (2017). The metabolic effects of GDF15 are mediated by the orphan receptor GFRAL. Nat. Med. 23, 1215–1219. doi: 10.1038/nm.4393, PMID: 28846098

[ref32] Englund A. Gilliam-Vigh H. Suppli M. P. Gasbjerg L. S. Vilsbøll T. Knop F. K. (2024). Intestinal expression profiles and hepatic expression of LEAP2, ghrelin and their common receptor, GHSR, in humans. Peptides 177:171227. doi: 10.1016/j.peptides.2024.171227, PMID: 38657907

[ref33] Erdem N. B. Kahramanoğlu Aksoy E. Dikmen D. Uçar Baş K. Ağaçdiken A. İlhan Esgin M.. (2024). Effects of low fat diet on inflammatory parameters in individuals with obesity/overweight and non-alcoholic fatty liver disease: a cross-sectional study. Medicine (Baltimore) 103:e37716. doi: 10.1097/MD.0000000000037716, PMID: 38608067 PMC11018204

[ref34] Espes D. Lau J. Carlsson P. O. (2014). Increased circulating levels of betatrophin in individuals with long-standing type 1 diabetes. Diabetologia 57, 50–53. doi: 10.1007/s00125-013-3071-1, PMID: 24078058 PMC3855541

[ref35] Fazeli P. K. Lun M. Kim S. M. Bredella M. A. Wright S. Zhang Y.. (2015). FGF21 and the late adaptive response to starvation in humans. J. Clin. Invest. 125, 4601–4611. doi: 10.1172/JCI83349, PMID: 26529252 PMC4665770

[ref36] Fernandez G. Cabral A. De Francesco P. N. Uriarte M. Reynaldo M. Castrogiovanni D.. (2022). GHSR controls food deprivation-induced activation of CRF neurons of the hypothalamic paraventricular nucleus in a LEAP2-dependent manner. Cell. Mol. Life Sci. 79:277. doi: 10.1007/s00018-022-04302-5, PMID: 35504998 PMC11072678

[ref37] Fisher F. M. Chui P. C. Antonellis P. J. Bina H. A. Kharitonenkov A. Flier J. S.. (2010). Obesity is a fibroblast growth factor 21 (FGF21)-resistant state. Diabetes 59, 2781–2789. doi: 10.2337/db10-0193, PMID: 20682689 PMC2963536

[ref38] Fittipaldi A. S. Hernández J. Castrogiovanni D. Lufrano D. De Francesco P. N. Garrido V.. (2020). Plasma levels of ghrelin, des-acyl ghrelin and LEAP2 in children with obesity: correlation with age and insulin resistance. Eur. J. Endocrinol. 182, 165–175. doi: 10.1530/EJE-19-0684, PMID: 31770106

[ref39] Frystyk J. Vestbo E. Skjaerbaek C. Mogensen C. E. Orskov H. (1995). Free insulin-like growth factors in human obesity. Metabolism 44, 37–44. doi: 10.1016/0026-0495(95)90219-8, PMID: 7476310

[ref40] Fu Z. Berhane F. Fite A. Seyoum B. Abou-Samra A. B. Zhang R. (2014). Elevated circulating lipasin/betatrophin in human type 2 diabetes and obesity. Sci. Rep. 4:5013. doi: 10.1038/srep05013, PMID: 24852694 PMC5381405

[ref41] Fu J. Malale K. Luo X. Chen M. Liu Q. Cheng W.. (2021). The relationship of mesencephalic astrocyte-derived neurotrophic factor with hyperlipidemia in patients with or without type 2 diabetes mellitus. Hormones (Athens) 20, 537–543. doi: 10.1007/s42000-021-00272-8, PMID: 33559083

[ref42] Gaich G. Chien J. Y. Fu H. Glass L. C. Deeg M. A. Holland W. L.. (2013). The effects of LY2405319, an FGF21 analog, in obese human subjects with type 2 diabetes. Cell Metab. 18, 333–340. doi: 10.1016/j.cmet.2013.08.005, PMID: 24011069

[ref43] Gao S. Ghoshal S. Zhang L. Stevens J. R. McCommis K. S. Finck B. N.. (2019). The peptide hormone adropin regulates signal transduction pathways controlling hepatic glucose metabolism in a mouse model of diet-induced obesity. J. Biol. Chem. 294, 13366–13377. doi: 10.1074/jbc.RA119.008967, PMID: 31324719 PMC6737218

[ref44] Gao S. McMillan R. P. Jacas J. Zhu Q. Li X. Kumar G. K.. (2014). Regulation of substrate oxidation preferences in muscle by the peptide hormone adropin. Diabetes 63, 3242–3252. doi: 10.2337/db14-0388, PMID: 24848071 PMC4171656

[ref45] Garcia-Luna C. Sanchez-Watts G. Arnold M. de Lartigue G. DeWalt N. Langhans W.. (2021). The medullary targets of Neurally conveyed sensory information from the rat hepatic portal and superior mesenteric veins. eNeuro 8. doi: 10.1523/ENEURO.0419-20.2021 PMC8114873 33495245

[ref46] Ge X. Yang H. Bednarek M. A. Galon-Tilleman H. Chen P. Chen M.. (2018). LEAP2 is an endogenous antagonist of the ghrelin receptor. Cell Metab. 27, 461–469.e6. doi: 10.1016/j.cmet.2017.10.016, PMID: 29233536

[ref47] Gharipour M. Sadeghi M. Salehi M. Behmanesh M. Khosravi E. Dianatkhah M.. (2017). Association of expression of selenoprotein P in mRNA and protein levels with metabolic syndrome in subjects with cardiovascular disease: results of the Selenegene study. J. Gene Med. 19. doi: 10.1002/jgm.2945, PMID: 28190280

[ref48] Ghodsian N. Gagnon E. Bourgault J. Gobeil É. Manikpurage H. D. Perrot N.. (2021). Blood levels of the SMOC1 Hepatokine are not causally linked with type 2 diabetes: a bidirectional Mendelian randomization study. Nutrients 13:4208. doi: 10.3390/nu13124208, PMID: 34959760 PMC8706295

[ref49] Griffin J. D. Buxton J. M. Culver J. A. Barnes R. Jordan E. A. White A. R.. (2023). Hepatic Activin E mediates liver-adipose inter-organ communication, suppressing adipose lipolysis in response to elevated serum fatty acids. Mol. Metab. 78:101830. doi: 10.1016/j.molmet.2023.101830, PMID: 38787338 PMC10656223

[ref50] Hagemann C. A. Jensen M. S. Holm S. Gasbjerg L. S. Byberg S. Skov-Jeppesen K.. (2022). LEAP2 reduces postprandial glucose excursions and ad libitum food intake in healthy men. Cell Rep. Med. 3:100582. doi: 10.1016/j.xcrm.2022.100582, PMID: 35492241 PMC9043997

[ref51] Hale C. Véniant M. M. (2021). Growth differentiation factor 15 as a potential therapeutic for treating obesity. Mol. Metab. 46:101117. doi: 10.1016/j.molmet.2020.101117, PMID: 33220493 PMC8085570

[ref52] Hansen J. Rinnov A. Krogh-Madsen R. Fischer C. P. Andreasen A. S. Berg R. M. G.. (2013). Plasma follistatin is elevated in patients with type 2 diabetes: relationship to hyperglycemia, hyperinsulinemia, and systemic low-grade inflammation. Diabetes Metab. Res. Rev. 29, 463–472. doi: 10.1002/dmrr.2415, PMID: 23564759

[ref53] Hashimoto O. Funaba M. Sekiyama K. Doi S. Shindo D. Satoh R.. (2018). Activin E controls energy homeostasis in both Brown and White adipose tissues as a Hepatokine. Cell Rep. 25, 1193–1203. doi: 10.1016/j.celrep.2018.10.008, PMID: 30380411

[ref54] Hes C. Gui L. T. Bay A. Alvarez F. Katz P. Paul T.. (2025). GDNF family receptor alpha-like (GFRAL) expression is restricted to the caudal brainstem. Mol. Metab. 91:102070. doi: 10.1016/j.molmet.2024.102070, PMID: 39608751 PMC11650321

[ref55] Holá L. Tureckiuová T. Kuneš J. Železná B. Maletínská L. (2023). High-fat diet induces resistance to ghrelin and LEAP2 peptide analogs in mice. Physiol. Res. 72, 607–619. doi: 10.33549/physiolres.935189, PMID: 38015760 PMC10751049

[ref56] Holá L. Železná B. Karnošová A. Kuneš J. Fehrentz J. A. Denoyelle S.. (2022). A novel truncated liver enriched antimicrobial Peptide-2 Palmitoylated at its N-terminal antagonizes effects of ghrelin. J. Pharmacol. Exp. Ther. 383, 129–136. doi: 10.1124/jpet.122.001322, PMID: 36198495

[ref57] Holm S. Husted A. S. Skov L. J. Morville T. H. Hagemann C. A. Jorsal T.. (2022). Beta-Hydroxybutyrate suppresses hepatic production of the ghrelin receptor antagonist LEAP2. Endocrinology 163. doi: 10.1210/endocr/bqac038, PMID: 35352108 PMC9119693

[ref58] Hong H. Cui Z. Z. Zhu L. Fu S. P. Rossi M. Cui Y. H.. (2017). Central IGF1 improves glucose tolerance and insulin sensitivity in mice. Nutr Diabetes 7, 1–10. doi: 10.1038/s41387-017-0002-0 29259155 PMC5865549

[ref59] Hsu J. Y. Crawley S. Chen M. Ayupova D. A. Lindhout D. A. Higbee J.. (2017). Non-homeostatic body weight regulation through a brainstem-restricted receptor for GDF15. Nature 550, 255–259. doi: 10.1038/nature24042, PMID: 28953886

[ref60] Hsuchou H. Pan W. Kastin A. J. (2007). The fasting polypeptide FGF21 can enter brain from blood. Peptides 28, 2382–2386. doi: 10.1016/j.peptides.2007.10.007, PMID: 17996984 PMC2151924

[ref61] Hu H. Sun W. Yu S. Hong X. Qian W. Tang B.. (2014). Increased circulating levels of betatrophin in newly diagnosed type 2 diabetic patients. Diabetes Care 37, 2718–2722. doi: 10.2337/dc14-0602 25024395

[ref62] Huang R. L. Li C. H. Du Y. F. Cheng K. P. Lin C. H. Hu C. Y.. (2020). Discovery of a role of the novel hepatokine, hepassocin, in obesity. Biofactors 46, 100–105. doi: 10.1002/biof.1574, PMID: 31587376

[ref63] Huffman D. M. Farias Quipildor G. Mao K. Zhang X. Wan J. Apontes P.. (2016). Central insulin-like growth factor-1 (IGF-1) restores whole-body insulin action in a model of age-related insulin resistance and IGF-1 decline. Aging Cell 15, 181–186. doi: 10.1111/acel.12415, PMID: 26534869 PMC4717281

[ref64] Islam M. N. Mita Y. Maruyama K. Tanida R. Zhang W. Sakoda H.. (2020). Liver-expressed antimicrobial peptide 2 antagonizes the effect of ghrelin in rodents. J. Endocrinol. 244, 13–23. doi: 10.1530/JOE-19-0102, PMID: 31539874 PMC6839046

[ref65] Islam M. N. Nabekura H. Ueno H. Nishida T. Nanashima A. Sakoda H.. (2024). Liver-expressed antimicrobial peptide 2 is a hepatokine regulated by ghrelin, nutrients, and body weight. Sci. Rep. 14:24782. doi: 10.1038/s41598-024-74048-6, PMID: 39433849 PMC11494003

[ref66] Jais A. Brüning J. C. (2022). Arcuate nucleus-dependent regulation of metabolism-pathways to obesity and diabetes mellitus. Endocr. Rev. 43, 314–328. doi: 10.1210/endrev/bnab025, PMID: 34490882 PMC8905335

[ref67] Janssen A. W. F. Katiraei S. Bartosinska B. Eberhard D. Willems van Dijk K. Kersten S. (2018). Loss of angiopoietin-like 4 (ANGPTL4) in mice with diet-induced obesity uncouples visceral obesity from glucose intolerance partly via the gut microbiota. Diabetologia 61, 1447–1458. doi: 10.1007/s00125-018-4583-5, PMID: 29502266 PMC6449003

[ref68] Jensen-Cody S. O. Potthoff M. J. (2020). Hepatokines and metabolism: deciphering communication from the liver. Molec. Metab. 44:101138. doi: 10.1016/j.molmet.2020.101138, PMID: 33285302 PMC7788242

[ref69] Kappeler L. Filho C. D. M. Dupont J. Leneuve P. Cervera P. Périn L.. (2008). Brain IGF-1 receptors control mammalian growth and lifespan through a neuroendocrine mechanism. PLoS Biol. 6:e254. doi: 10.1371/journal.pbio.0060254, PMID: 18959478 PMC2573928

[ref70] Kersten S. (2021). ANGPTL3 as therapeutic target. Curr. Opin. Lipidol. 32, 335–341. doi: 10.1097/MOL.0000000000000789, PMID: 34581310 PMC8631155

[ref71] Kharitonenkov A. Shiyanova T. L. Koester A. Ford A. M. Micanovic R. Galbreath E. J.. (2005). FGF-21 as a novel metabolic regulator. J. Clin. Invest. 115, 1627–1635. doi: 10.1172/JCI23606, PMID: 15902306 PMC1088017

[ref72] Kim H. K. Youn B. S. Shin M. S. Namkoong C. Park K. H. Baik J. H.. (2010). Hypothalamic Angptl4/Fiaf is a novel regulator of food intake and body weight. Diabetes 59, 2772–2780. doi: 10.2337/db10-0145, PMID: 20798332 PMC2963535

[ref73] Koishi R. Ando Y. Ono M. Shimamura M. Yasumo H. Fujiwara T.. (2002). Angptl3 regulates lipid metabolism in mice. Nat. Genet. 30, 151–157. doi: 10.1038/ng814 11788823

[ref74] Koo B. K. Um S. H. Seo D. S. Joo S. K. Bae J. M. Park J. H.. (2018). Growth differentiation factor 15 predicts advanced fibrosis in biopsy-proven non-alcoholic fatty liver disease. Liver Int. 38, 695–705. doi: 10.1111/liv.13587, PMID: 28898507

[ref75] Köster A. Chao Y. B. Mosior M. Ford A. Gonzalez-DeWhitt P. A. Hale J. E.. (2005). Transgenic angiopoietin-like (angptl)4 overexpression and targeted disruption of angptl4 and angptl3: regulation of triglyceride metabolism. Endocrinology 146, 4943–4950. doi: 10.1210/en.2005-0476, PMID: 16081640

[ref76] Krause A. Sillard R. Kleemeier B. Klüver E. Maronde E. Conejo-García J. R.. (2003). Isolation and biochemical characterization of LEAP-2, a novel blood peptide expressed in the liver. Protein Sci. 12, 143–152. doi: 10.1110/ps.0213603, PMID: 12493837 PMC2312392

[ref77] Kumar K. G. Trevaskis J. L. Lam D. D. Sutton G. M. Koza R. A. Chouljenko V. N.. (2008). Identification of adropin as a secreted factor linking dietary macronutrient intake with energy homeostasis and lipid metabolism. Cell Metab. 8, 468–481. doi: 10.1016/j.cmet.2008.10.011, PMID: 19041763 PMC2746325

[ref78] Kutlu O. Altun Ö. Dikker O. Aktaş Ş. Özsoy N. Arman Y.. (2019). Serum Adropin levels are reduced in adult patients with nonalcoholic fatty liver disease. Med. Princ. Pract. 28, 463–469. doi: 10.1159/000500106, PMID: 30995640 PMC6771072

[ref79] Kwon E. Joung H. Y. Liu S. M. Chua S. C. Schwartz G. J. Jo Y. H. (2020). Optogenetic stimulation of the liver-projecting melanocortinergic pathway promotes hepatic glucose production. Nat. Commun. 11:6295. doi: 10.1038/s41467-020-20160-w, PMID: 33293550 PMC7722761

[ref80] Lam S. Lee C. H. Fong C. H. Y. Wong Y. Shiu S. W. M. Mak L. Y.. (2024). Serum Tsukushi level is associated with the severity of liver fibrosis independent of type 2 diabetes. J. Clin. Endocrinol. Metab. 109, e1048–e1054. doi: 10.1210/clinem/dgad650, PMID: 37933700

[ref81] Lan F. Misu H. Chikamoto K. Takayama H. Kikuchi A. Mohri K.. (2014). LECT2 functions as a Hepatokine that links obesity to skeletal muscle insulin resistance. Diabetes 63, 1649–1664. doi: 10.2337/db13-0728, PMID: 24478397

[ref82] Lee Y. H. Lee S. G. Lee C. J. Kim S. H. Song Y. M. Yoon M. R.. (2016). Association between betatrophin/ANGPTL8 and non-alcoholic fatty liver disease: animal and human studies. Sci. Rep. 6:24013. doi: 10.1038/srep24013, PMID: 27045862 PMC4820743

[ref83] Li Y. Deng X. Wu X. Zhao L. Zhao Z. Guo C.. (2023). Association of serum Tsukushi level with metabolic syndrome and its components. Endocrine 79, 469–476. doi: 10.1007/s12020-022-03285-4, PMID: 36592295

[ref84] Li J. Huang P. Xiong J. Liang X. Li M. Ke H.. (2022). Serum levels of ghrelin and LEAP2 in patients with type 2 diabetes mellitus: correlation with circulating glucose and lipids. Endocr. Connect. 11:e220012. doi: 10.1530/EC-22-0012, PMID: 35521798 PMC9175609

[ref85] Li Y. Jin L. Yan J. Huang Y. Zhang H. Zhang R.. (2021). Tsukushi and TSKU genotype in obesity and related metabolic disorders. J. Endocrinol. Investig. 44, 2645–2654. doi: 10.1007/s40618-021-01572-x, PMID: 33860453 PMC8572186

[ref86] Li Q. Yu Y. Kang Y. Xu J. Lin H. Wang X.. (2016). Correlations of cerebrospinal fluid/plasma fibroblast growth factor 21 ratio with metabolic parameters in Chinese individuals of Normal weight. Clin. Lab. 62, 893–899. doi: 10.7754/Clin.Lab.2015.150926, PMID: 27349016

[ref87] Li H. Zhang C. Liu J. Xie W. Xu W. Liang F.. (2019). Intraperitoneal administration of follistatin promotes adipocyte browning in high-fat diet-induced obese mice. PLoS One 14:e0220310. doi: 10.1371/journal.pone.0220310, PMID: 31365569 PMC6668797

[ref88] Liang Q. Zhong L. Zhang J. Wang Y. Bornstein S. R. Triggle C. R.. (2014). FGF21 maintains glucose homeostasis by mediating the cross talk between liver and brain during prolonged fasting. Diabetes 63, 4064–4075. doi: 10.2337/db14-0541, PMID: 25024372

[ref89] Lindahl M. Danilova T. Palm E. Lindholm P. Võikar V. Hakonen E.. (2014). MANF is indispensable for the proliferation and survival of pancreatic β cells. Cell Rep. 7, 366–375. doi: 10.1016/j.celrep.2014.03.023, PMID: 24726366 PMC7254957

[ref90] Lockhart S. M. Saudek V. O’Rahilly S. (2020). GDF15: a hormone conveying somatic distress to the brain. Endocr. Rev. 41. doi: 10.1210/endrev/bnaa007, PMID: 32310257 PMC7299427

[ref91] Lugilde J. Casado S. Beiroa D. Cuñarro J. Garcia-Lavandeira M. Álvarez C. V.. (2022). LEAP-2 counteracts ghrelin-induced food intake in a nutrient, growth hormone and age independent manner. Cells 11:324. doi: 10.3390/cells11030324, PMID: 35159134 PMC8834077

[ref92] Luo M. Peng D. (2018). ANGPTL8: an important regulator in metabolic disorders. Front Endocrinol. 9:169. doi: 10.3389/fendo.2018.00169, PMID: 29719529 PMC5913278

[ref93] M’Kadmi C. Cabral A. Barrile F. Giribaldi J. Cantel S. Damian M.. (2019). N-terminal liver-expressed antimicrobial peptide 2 (LEAP2) region exhibits inverse agonist activity toward the ghrelin receptor. J. Med. Chem. 62, 965–973. doi: 10.1021/acs.jmedchem.8b01644, PMID: 30543423

[ref94] Maekawa R. Seino Y. Ogata H. Murase M. Iida A. Hosokawa K.. (2017). Chronic high-sucrose diet increases fibroblast growth factor 21 production and energy expenditure in mice. J. Nutr. Biochem. 49, 71–79. doi: 10.1016/j.jnutbio.2017.07.010, PMID: 28886439

[ref95] Maïmoun L. Mura T. Attalin V. Dupuy A. M. Cristol J. P. Avignon A.. (2020). Modification of muscle-related hormones in women with obesity: potential impact on bone metabolism. J. Clin. Med. 9:1150. doi: 10.3390/jcm9041150, PMID: 32316563 PMC7230770

[ref96] Mani B. K. Puzziferri N. He Z. Rodriguez J. A. Osborne-Lawrence S. Metzger N. P.. (2019). LEAP2 changes with body mass and food intake in humans and mice. J. Clin. Invest. 129, 3909–3923. doi: 10.1172/JCI125332, PMID: 31424424 PMC6715358

[ref97] Mani B. K. Shankar K. Zigman J. M. (2019). Ghrelin’s relationship to blood glucose. Endocrinology 160, 1247–1261. doi: 10.1210/en.2019-00074, PMID: 30874792 PMC6482034

[ref98] Markan K. R. Naber M. C. Ameka M. K. Anderegg M. D. Mangelsdorf D. J. Kliewer S. A.. (2014). Circulating FGF21 is liver derived and enhances glucose uptake during refeeding and overfeeding. Diabetes 63, 4057–4063. doi: 10.2337/db14-0595, PMID: 25008183 PMC4238010

[ref99] Mathews S. T. Singh G. P. Ranalletta M. Cintron V. J. Qiang X. Goustin A. S.. (2002). Improved insulin sensitivity and resistance to weight gain in mice null for the Ahsg gene. Diabetes 51, 2450–2458. doi: 10.2337/diabetes.51.8.2450, PMID: 12145157

[ref100] McCulloch L. J. Bramwell L. R. Knight B. Kos K. (2020). Circulating and tissue specific transcription of angiopoietin-like protein 4 in human type 2 diabetes. Metabolism 106:154192. doi: 10.1016/j.metabol.2020.154192, PMID: 32112823

[ref101] Meng X. Li D. Kan R. Xiang Y. Pan L. Guo Y.. (2024). Inhibition of ANGPTL8 protects against diabetes-associated cognitive dysfunction by reducing synaptic loss via the PirB signaling pathway. J. Neuroinflammation 21:192. doi: 10.1186/s12974-024-03183-8, PMID: 39095838 PMC11297729

[ref102] Misu H. Takamura T. Takayama H. Hayashi H. Matsuzawa-Nagata N. Kurita S.. (2010). A liver-derived secretory protein, selenoprotein P, causes insulin resistance. Cell Metab. 12, 483–495. doi: 10.1016/j.cmet.2010.09.015, PMID: 21035759

[ref103] Montgomery M. K. Bayliss J. Devereux C. Bezawork-Geleta A. Roberts D. Huang C.. (2020). SMOC1 is a glucose-responsive hepatokine and therapeutic target for glycemic control. Sci. Transl. Med. 12. doi: 10.1126/scitranslmed.aaz8048, PMID: 32878981

[ref104] Mouchiroud M. Camiré É. Aldow M. Caron A. Jubinville É. Turcotte L.. (2019a). The Hepatokine TSK does not affect brown fat thermogenic capacity, body weight gain, and glucose homeostasis. Molec. Metab. 30, 184–191. doi: 10.1016/j.molmet.2019.09.014, PMID: 31767170 PMC6889588

[ref105] Mouchiroud M. Camiré É. Aldow M. Caron A. Jubinville É. Turcotte L.. (2019b). The hepatokine Tsukushi is released in response to NAFLD and impacts cholesterol homeostasis. JCI Insight 4:129492. doi: 10.1172/jci.insight.129492 31391339 PMC6693835

[ref106] Mullican S. E. Lin-Schmidt X. Chin C. N. Chavez J. A. Furman J. L. Armstrong A. A.. (2017). GFRAL is the receptor for GDF15 and the ligand promotes weight loss in mice and nonhuman primates. Nat. Med. 23, 1150–1157. doi: 10.1038/nm.4392, PMID: 28846097

[ref107] Muzumdar R. H. Ma X. Fishman S. Yang X. Atzmon G. Vuguin P.. (2006). Central and opposing effects of IGF-I and IGF-binding Protein-3 on systemic insulin action. Diabetes 55, 2788–2796. doi: 10.2337/db06-0318, PMID: 17003344

[ref108] Namkung J. Koh S. B. Kong I. D. Choi J. W. Yeh B. I. (2011). Serum levels of angiopoietin-related growth factor are increased in metabolic syndrome. Metabolism 60, 564–568. doi: 10.1016/j.metabol.2010.05.013, PMID: 20673930

[ref109] Ogawa Y. Kurosu H. Yamamoto M. Nandi A. Rosenblatt K. P. Goetz R.. (2007). BetaKlotho is required for metabolic activity of fibroblast growth factor 21. Proc. Natl. Acad. Sci. USA 104, 7432–7437. doi: 10.1073/pnas.0701600104, PMID: 17452648 PMC1855074

[ref110] Oike Y. Akao M. Yasunaga K. Yamauchi T. Morisada T. Ito Y.. (2005). Angiopoietin-related growth factor antagonizes obesity and insulin resistance. Nat. Med. 11, 400–408. doi: 10.1038/nm1214 15778720

[ref111] Olshan D. S. Rader D. J. (2018). Angiopoietin-like protein 4: a therapeutic target for triglycerides and coronary disease? J. Clin. Lipidol. 12, 583–587. doi: 10.1016/j.jacl.2018.01.012, PMID: 29548670

[ref112] Ou H. Y. Wu H. T. Lin C. H. Du Y. F. Hu C. Y. Hung H. C.. (2017). The hepatic protection effects of Hepassocin in hyperglycemic crisis. J. Clin. Endocrinol. Metab. 102, 2407–2415. doi: 10.1210/jc.2016-3287, PMID: 28402540

[ref113] Owen B. M. Ding X. Morgan D. A. Coate K. C. Bookout A. L. Rahmouni K.. (2014). FGF21 acts centrally to induce sympathetic nerve activity, energy expenditure and weight loss. Cell Metab. 20, 670–677. doi: 10.1016/j.cmet.2014.07.012, PMID: 25130400 PMC4192037

[ref114] Pakarinen E. Danilova T. Võikar V. Chmielarz P. Piepponen P. Airavaara M.. (2020). MANF ablation causes prolonged activation of the UPR without neurodegeneration in the mouse midbrain dopamine system. eNeuro 7, ENEURO.0477–ENEU19.2019. doi: 10.1523/ENEURO.0477-19.2019, PMID: 32005751 PMC7053174

[ref115] Patel S. Alvarez-Guaita A. Melvin A. Rimmington D. Dattilo A. Miedzybrodzka E. L.. (2019). GDF15 provides an endocrine signal of nutritional stress in mice and humans. Cell Metab. 29, 707–718.e8. doi: 10.1016/j.cmet.2018.12.016, PMID: 30639358 PMC6408327

[ref116] Perelló M. (2025). Critical insights into LEAP2 biology and physiological functions: potential roles beyond ghrelin antagonism. Endocrinology 166. doi: 10.1210/endocr/bqaf011 39823403

[ref117] Qaddoumi M. G. Alanbaei M. Hammad M. M. Al Khairi I. Cherian P. Channanath A.. (2020). Investigating the role of myeloperoxidase and angiopoietin-like protein 6 in obesity and diabetes. Sci. Rep. 10:6170. doi: 10.1038/s41598-020-63149-7, PMID: 32277104 PMC7148302

[ref118] Quagliarini F. Wang Y. Kozlitina J. Grishin N. V. Hyde R. Boerwinkle E.. (2012). Atypical angiopoietin-like protein that regulates ANGPTL3. Proc. Natl. Acad. Sci. USA 109, 19751–19756. doi: 10.1073/pnas.1217552109, PMID: 23150577 PMC3511699

[ref119] Ragland T. J. Malin S. K. (2023). Plasma LEAP-2 following a low-calorie diet with or without interval exercise in women with obesity. Nutrients 15:655. doi: 10.3390/nu15030655, PMID: 36771362 PMC9918887

[ref120] Rahmani J. Kord Varkaneh H. Clark C. Zand H. Bawadi H. Ryan P. M.. (2019). The influence of fasting and energy restricting diets on IGF-1 levels in humans: a systematic review and meta-analysis. Ageing Res. Rev. 53:100910. doi: 10.1016/j.arr.2019.100910, PMID: 31116995

[ref121] Rawal S. U. Patel B. M. Patel M. M. (2022). New drug delivery systems developed for brain targeting. Drugs 82, 749–792. doi: 10.1007/s40265-022-01717-z, PMID: 35596879

[ref122] Saez-Lopez C. Villena J. A. Simó R. Selva D. M. (2020). Sex hormone-binding globulin overexpression protects against high-fat diet-induced obesity in transgenic male mice. J. Nutr. Biochem. 85:108480. doi: 10.1016/j.jnutbio.2020.108480, PMID: 32795655

[ref123] Sarruf D. A. Thaler J. P. Morton G. J. German J. Fischer J. D. Ogimoto K.. (2010). Fibroblast growth factor 21 action in the brain increases energy expenditure and insulin sensitivity in obese rats. Diabetes 59, 1817–1824. doi: 10.2337/db09-1878, PMID: 20357365 PMC2889784

[ref124] Schinzari F. Vizioli G. Campia U. Tesauro M. Cardillo C. (2021). Variable changes of circulating ANGPTL3 and ANGPTL4 in different obese phenotypes: relationship with vasodilator dysfunction. Biomedicines 9:1037. doi: 10.3390/biomedicines9081037, PMID: 34440242 PMC8393675

[ref125] Schnurr T. M. Jakupović H. Carrasquilla G. D. Ängquist L. Grarup N. Sørensen T. I. A.. (2020). Obesity, unfavourable lifestyle and genetic risk of type 2 diabetes: a case-cohort study. Diabetologia 63, 1324–1332. doi: 10.1007/s00125-020-05140-5, PMID: 32291466

[ref126] Schulze R. J. Schott M. B. Casey C. A. Tuma P. L. McNiven M. A. (2019). The cell biology of the hepatocyte: a membrane trafficking machine. J. Cell Biol. 218, 2096–2112. doi: 10.1083/jcb.201903090, PMID: 31201265 PMC6605791

[ref127] Sekiyama K. Ushiro Y. Kurisaki A. Funaba M. Hashimoto O. (2019). Activin E enhances insulin sensitivity and thermogenesis by activating brown/beige adipocytes. J. Vet. Med. Sci. 81, 646–652. doi: 10.1292/jvms.19-0036, PMID: 30880304 PMC6541856

[ref128] Shankar K. Metzger N. P. Singh O. Mani B. K. Osborne-Lawrence S. Varshney S.. (2021). LEAP2 deletion in mice enhances ghrelin’s actions as an orexigen and growth hormone secretagogue. Mol. Metab. 53:101327. doi: 10.1016/j.molmet.2021.101327, PMID: 34428557 PMC8452786

[ref129] Sheng L. Cho K. W. Zhou Y. Shen H. Rui L. (2011). Lipocalin 13 protein protects against hepatic steatosis by both inhibiting lipogenesis and stimulating fatty acid β-oxidation. J. Biol. Chem. 286, 38128–38135. doi: 10.1074/jbc.M111.256677, PMID: 21908604 PMC3207413

[ref130] Shin J. Y. Kim S. K. Lee M. Y. Kim H. S. Ye B. I. Shin Y. G.. (2011). Serum sex hormone-binding globulin levels are independently associated with nonalcoholic fatty liver disease in people with type 2 diabetes. Diabetes Res. Clin. Pract. 94, 156–162. doi: 10.1016/j.diabres.2011.07.029, PMID: 21862168

[ref131] Singh R. Braga M. Reddy S. T. Lee S. J. Parveen M. Grijalva V.. (2017). Follistatin targets distinct pathways To promote Brown adipocyte characteristics in Brown and White adipose tissues. Endocrinology 158, 1217–1230. doi: 10.1210/en.2016-1607, PMID: 28324027 PMC5460830

[ref132] Singh A. K. Chaube B. Zhang X. Sun J. Citrin K. M. Canfrán-Duque A.. (2021). Hepatocyte-specific suppression of ANGPTL4 improves obesity-associated diabetes and mitigates atherosclerosis in mice. J. Clin. Invest. 131:e140989. doi: 10.1172/JCI140989, PMID: 34255741 PMC8409581

[ref133] Sousa-Victor P. Neves J. Cedron-Craft W. Ventura P. B. Liao C. Y. Riley R. R.. (2019). MANF regulates metabolic and immune homeostasis in ageing and protects against liver damage. Nat. Metab. 1, 276–290. doi: 10.1038/s42255-018-0023-6, PMID: 31489403 PMC6727652

[ref134] Spolcová A. Holubová M. Mikulášková B. Nagelová V. Stofková A. Lacinová Z.. (2014). Changes in FGF21 serum concentrations and liver mRNA expression in an experimental model of complete lipodystrophy and insulin-resistant diabetes. Physiol. Res. 63, 483–490. doi: 10.33549/physiolres.932714, PMID: 24908095

[ref135] Stanley S. Pinto S. Segal J. Pérez C. A. Viale A. DeFalco J.. (2010). Identification of neuronal subpopulations that project from hypothalamus to both liver and adipose tissue polysynaptically. Proceed. Natl. Acad. Sci. U. S. A. 107, 7024–7029. doi: 10.1073/pnas.1002790107 PMC2872469 20351287

[ref136] Stark R. Feehan J. Mousa A. Andrews Z. B. de Courten B. (2023). Liver-expressed antimicrobial peptide 2 is associated with improved pancreatic insulin secretion in adults with overweight and obesity. Diabetes Obes. Metab. 25, 1213–1220. doi: 10.1111/dom.14968, PMID: 36597795 PMC10947148

[ref137] Straus D. S. Takemoto C. D. (1990). Effect of fasting on insulin-like growth factor-I (IGF-I) and growth hormone receptor mRNA levels and IGF-I gene transcription in rat liver. Mol. Endocrinol. 4, 91–100. doi: 10.1210/mend-4-1-91, PMID: 2325671

[ref138] Sugiyama M. Kikuchi A. Misu H. Igawa H. Ashihara M. Kushima Y.. (2018). Inhibin βE (INHBE) is a possible insulin resistance-associated hepatokine identified by comprehensive gene expression analysis in human liver biopsy samples. PLoS One 13:e0194798. doi: 10.1371/journal.pone.0194798, PMID: 29596463 PMC5875797

[ref139] Sylow L. Vind B. F. Kruse R. Møller P. M. Wojtaszewski J. F. P. Richter E. A.. (2020). Circulating Follistatin and Activin a and their regulation by insulin in obesity and type 2 diabetes. J. Clin. Endocrinol. Metab. 105, 1343–1354. doi: 10.1210/clinem/dgaa090, PMID: 32112102

[ref140] Sylvers-Davie K. L. Davies B. S. J. (2021). Regulation of lipoprotein metabolism by ANGPTL3, ANGPTL4, and ANGPTL8. Am. J. Physiol. Endocrinol. Metab. 321, E493–E508. doi: 10.1152/ajpendo.00195.2021, PMID: 34338039 PMC8560382

[ref141] Tan B. K. Hallschmid M. Adya R. Kern W. Lehnert H. Randeva H. S. (2011). Fibroblast growth factor 21 (FGF21) in human cerebrospinal fluid: relationship with plasma FGF21 and body adiposity. Diabetes 60, 2758–2762. doi: 10.2337/db11-0672, PMID: 21926274 PMC3198100

[ref142] Tan B. K. Sivakumar K. Bari M. F. Vatish M. Randeva H. S. (2013). Lower cerebrospinal fluid/plasma fibroblast growth factor 21 (FGF21) ratios and placental FGF21 production in gestational diabetes. PLoS One 8:e65254. doi: 10.1371/journal.pone.0065254, PMID: 23755203 PMC3670883

[ref143] Tang Q. Li Y. He J. (2022). MANF: an emerging therapeutic target for metabolic diseases. Trends Endocrinol. Metab. 33, 236–246. doi: 10.1016/j.tem.2022.01.001, PMID: 35135706

[ref144] Tong J. Cong L. Jia Y. He B. L. Guo Y. He J.. (2022). Follistatin alleviates hepatic steatosis in NAFLD via the mTOR dependent pathway. Diabetes Metab. Syndr. Obes. 15, 3285–3301. doi: 10.2147/DMSO.S380053, PMID: 36325432 PMC9621035

[ref145] Tsai V. W. W. Manandhar R. Jørgensen S. B. Lee-Ng K. K. M. Zhang H. P. Marquis C. P.. (2014). The anorectic actions of the TGFβ cytokine MIC-1/GDF15 require an intact brainstem area Postrema and nucleus of the solitary tract. PLoS One 9:e100370. doi: 10.1371/journal.pone.0100370, PMID: 24971956 PMC4074070

[ref146] Tufvesson-Alm M. Zhang Q. Aranäs C. Sköldheden S. B. Edvardsson C. E. Jerlhag E. (2023). Decoding the influence of central LEAP2 on hedonic food intake and its association with dopaminergic reward pathways [internet]. bioRxiv. doi: 10.1038/s41398-024-03136-y

[ref147] Uhlén M. Fagerberg L. Hallström B. M. Lindskog C. Oksvold P. Mardinoglu A.. (2015). Tissue-based map of the human proteome. Science 347:1260419. doi: 10.1126/science.1260419, PMID: 25613900

[ref148] Vienberg S. G. Kleinridders A. Suzuki R. Kahn C. R. (2015). Differential effects of angiopoietin-like 4 in brain and muscle on regulation of lipoprotein lipase activity. Mol. Metab. 4, 144–150. doi: 10.1016/j.molmet.2014.11.003, PMID: 25685701 PMC4314546

[ref149] von Loeffelholz C. Pfeiffer A. F. H. Lock J. F. Lieske S. Döcke S. Murahovschi V.. (2017). ANGPTL8 (Betatrophin) is expressed in visceral adipose tissue and relates to human hepatic steatosis in two independent clinical collectives. Horm. Metab. Res. 49, 343–349. doi: 10.1055/s-0043-102950, PMID: 28351093

[ref150] Wang D. Day E. A. Townsend L. K. Djordjevic D. Jørgensen S. B. Steinberg G. R. (2021). GDF15: emerging biology and therapeutic applications for obesity and cardiometabolic disease. Nat. Rev. Endocrinol. 17, 592–607. doi: 10.1038/s41574-021-00529-7, PMID: 34381196

[ref151] Wang M. Pugh S. M. Daboul J. Miller D. Xu Y. Hill J. W. (2024). IGF-1 acts through Kiss1-expressing cells to influence metabolism and reproduction. bioRxiv. doi: 10.1101/2024.07.02.601722

[ref152] Wang R. Yuan J. Zhang C. Wang L. Liu Y. Song L.. (2018). Neuropeptide Y-positive neurons in the dorsomedial hypothalamus are involved in the anorexic effect of Angptl8. Front. Mol. Neurosci. 11:451. doi: 10.3389/fnmol.2018.00451 30618603 PMC6305345

[ref153] Wiesner G. Morash B. A. Ur E. Wilkinson M. (2004). Food restriction regulates adipose-specific cytokines in pituitary gland but not in hypothalamus. J. Endocrinol. 180, R1–R6. doi: 10.1677/joe.0.180r001, PMID: 15012606

[ref154] Worth A. A. Shoop R. Tye K. Feetham C. H. D’Agostino G. Dodd G. T.. (2020). The cytokine GDF15 signals through a population of brainstem cholecystokinin neurons to mediate anorectic signalling. eLife 9:e55164. doi: 10.7554/eLife.55164, PMID: 32723474 PMC7410488

[ref155] Wu T. Liu Q. Li Y. Li H. Chen L. Yang X.. (2021). Feeding-induced hepatokine, Manf, ameliorates diet-induced obesity by promoting adipose browning via p38 MAPK pathway. J. Exp. Med. 218:e20201203. doi: 10.1084/jem.20201203, PMID: 33856409 PMC8054200

[ref156] Wu H. T. Lu F. H. Ou H. Y. Su Y. C. Hung H. C. Wu J. S.. (2013). The role of hepassocin in the development of non-alcoholic fatty liver disease. J. Hepatol. 59, 1065–1072. doi: 10.1016/j.jhep.2013.06.004, PMID: 23792031

[ref157] Xiong Y. Walker K. Min X. Hale C. Tran T. Komorowski R.. (2017). Long-acting MIC-1/GDF15 molecules to treat obesity: evidence from mice to monkeys. Sci. Transl. Med. 9. doi: 10.1126/scitranslmed.aan8732, PMID: 29046435

[ref158] Xiong X. Wang Q. Wang S. Zhang J. Liu T. Guo L.. (2019). Mapping the molecular signatures of diet-induced NASH and its regulation by the hepatokine Tsukushi. Mol Metab. 20, 128–137. doi: 10.1016/j.molmet.2018.12.004, PMID: 30595550 PMC6358550

[ref159] Yakar S. Liu J. L. Fernandez A. M. Wu Y. Schally A. V. Frystyk J.. (2001). Liver-specific igf-1 gene deletion leads to muscle insulin insensitivity. Diabetes 50, 1110–1118. doi: 10.2337/diabetes.50.5.1110, PMID: 11334415

[ref160] Yakar S. Setser J. Zhao H. Stannard B. Haluzik M. Glatt V.. (2004). Inhibition of growth hormone action improves insulin sensitivity in liver IGF-1-deficient mice. J. Clin. Invest. 113, 96–105. doi: 10.1172/JCI200417763, PMID: 14702113 PMC300761

[ref161] Yang L. Chang C. C. Sun Z. Madsen D. Zhu H. Padkjær S. B.. (2017). GFRAL is the receptor for GDF15 and is required for the anti-obesity effects of the ligand. Nat. Med. 23, 1158–1166. doi: 10.1038/nm.4394, PMID: 28846099

[ref162] Yang S. J. Hwang S. Y. Choi H. Y. Yoo H. J. Seo J. A. Kim S. G.. (2011). Serum Selenoprotein P levels in patients with type 2 diabetes and prediabetes: implications for insulin resistance, inflammation, and atherosclerosis. J. Clin. Endocrinol. Metabol. 96, E1325–E1329. doi: 10.1210/jc.2011-0620, PMID: 21677040

[ref163] Yang C. Jin C. Li X. Wang F. McKeehan W. L. Luo Y. (2012). Differential specificity of endocrine FGF19 and FGF21 to FGFR1 and FGFR4 in complex with KLB. PLoS One 7:e33870. doi: 10.1371/journal.pone.0033870, PMID: 22442730 PMC3307775

[ref164] Yoo H. J. Hwang S. Y. Choi J. H. Lee H. J. Chung H. S. Seo J. A.. (2017). Association of leukocyte cell-derived chemotaxin 2 (LECT2) with NAFLD, metabolic syndrome, and atherosclerosis. PLoS One 12:e0174717. doi: 10.1371/journal.pone.0174717, PMID: 28376109 PMC5380318

[ref165] Younossi Z. M. Koenig A. B. Abdelatif D. Fazel Y. Henry L. Wymer M. (2016). Global epidemiology of nonalcoholic fatty liver disease—Meta-analytic assessment of prevalence, incidence, and outcomes. Hepatology 64, 73–84. doi: 10.1002/hep.28431, PMID: 26707365

[ref166] Yuen K. C. J. Hjortebjerg R. Ganeshalingam A. A. Clemmons D. R. Frystyk J. (2024). Growth hormone/insulin-like growth factor I axis in health and disease states: an update on the role of intra-portal insulin. Front Endocrinol (Lausanne). 15:1456195. doi: 10.3389/fendo.2024.1456195, PMID: 39665021 PMC11632222

[ref167] Zang H. Jiang F. Cheng X. Xu H. Hu X. (2018). Serum adropin levels are decreased in Chinese type 2 diabetic patients and negatively correlated with body mass index. Endocr. J. 65, 685–691. doi: 10.1507/endocrj.EJ18-0060, PMID: 29669965

[ref168] Zhang R. (2012). Lipasin, a novel nutritionally-regulated liver-enriched factor that regulates serum triglyceride levels. Biochem. Biophys. Res. Commun. 424, 786–792. doi: 10.1016/j.bbrc.2012.07.038, PMID: 22809513

[ref169] Zhang Y. Wang Y. Liu J. (2023). Friend or foe for obesity: how hepatokines remodel adipose tissues and translational perspective. Genes Diseases 10, 825–847. doi: 10.1016/j.gendis.2021.12.011, PMID: 37396511 PMC10308077

[ref170] Zhang Z. Wu H. Dai L. Yuan Y. Zhu Y. Ma Z.. (2020). ANGPTL8 enhances insulin sensitivity by directly activating insulin-mediated AKT phosphorylation. Gene 749:144707. doi: 10.1016/j.gene.2020.144707, PMID: 32344005

[ref171] Zhang X. Yeung D. C. Y. Karpisek M. Stejskal D. Zhou Z. G. Liu F.. (2008). Serum FGF21 levels are increased in obesity and are independently associated with the metabolic syndrome in humans. Diabetes 57, 1246–1253. doi: 10.2337/db07-1476, PMID: 18252893

[ref172] Zhang Z. Zeng H. Lin J. Hu Y. Yang R. Sun J.. (2018). Circulating LECT2 levels in newly diagnosed type 2 diabetes mellitus and their association with metabolic parameters: an observational study. Medicine (Baltimore) 97:e0354. doi: 10.1097/MD.0000000000010354, PMID: 29642178 PMC5908589

[ref173] Zsombok A. Desmoulins L. D. Derbenev A. V. (2024). Sympathetic circuits regulating hepatic glucose metabolism: where we stand. Physiol. Rev. 104, 85–101. doi: 10.1152/physrev.00005.2023, PMID: 37440208 PMC11281813

